# Supplementary material for: Empathy and redemption: Exploring the narrative transformation of online support for mental health across communities before and after Covid-19
Source: PLoS One. 2024 Jul 25;19(7):e0307042. doi: 10.1371/journal.pone.0307042 (PMC11271960; doi:10.1371/journal.pone.0307042)
Supplement: S1 File — (PDF) [file pone.0307042.s001.pdf]

S1_File. Data Source Supporting Information

Our data were obtained from The-Eye.eu/redarcs, an open library of the Pushshift Reddit Dataset, which is building via the Pushshift Reddit API (https://github.com/pushshift/api) [5]. This database is widely used for nearly 1,000 of papers and API is using by widely research on Reddit communities [1,6]. Furthermore, all analyses and data did not involve personal information such as user IDs, adhering to data use policies [2]. Reddit allows moderators, researchers, developers, and other authorized users to access public data content for research purposes via API or third-party services. Reddit's official API is free and public and can be made available to third parties [3,4,5]

The extraction and cleaning of posts and comments from Reddit were primarily conducted using the NLTK tool kit in Python and using fine tunned Bert model from hugging face for sentiment analysis.

The text of Reddit posts and comments consists of user-generated content. In line with data privacy and ethical considerations, we don't extract user id and have anonymized other inforamation to safeguard user identity. According to its privacy policy (https://www.reddit.com/policies/privacy-policy?rdt=64144), Reddit permits third parties to access public content via the Reddit API and similar technologies. Thus, our data collection and sharing practices comply with Reddit's terms and conditions.

For access to the minimally anonymized dataset, please use this link: https://zenodo.org/records/12736756. The data's DOI is: 10.5281/zenodo.12736756[7].

1. Baumgartner J, Zannettou S, Keegan B, Squire M, Blackburn J. The Pushshift Reddit Dataset [Internet]. arXiv; 2020 [cited 2024 Jun 24]. Available from: http://arxiv.org/abs/2001.08435
2. Reddit Help [Internet]. 2024 [cited 2024 Jun 24]. Public Content Policy. Available from: https://support.reddithelp.com/hc/en-us/articles/26410290525844-Public-Content-Policy
3. Proferes N, Jones N, Gilbert S, Fiesler C, Zimmer M. Studying Reddit: A Systematic Overview of Disciplines, Approaches, Methods, and Ethics. Social Media + Society. 2021 Apr 1;7(2):20563051211019004.
4. Gaudette T, Scrivens R, Davies G, Frank R. Upvoting extremism: Collective identity formation and the extreme right on Reddit. New Media & Society. 2021 Dec 1;23(12):3491–508.
5. Baumgartner JM. pushshift/api [Internet]. 2024 [cited 2024 Jun 25]. Available from: https://github.com/pushshift/api
6. Gaudette T, Scrivens R, Davies G, Frank R. Upvoting extremism: Collective identity formation and the extreme right on Reddit. New Media & Society. 2021 Dec 1;23(12):3491–508.
7. 1. Wei E, Cai Y. Supportive behavior of the reddit community [Internet]. Zenodo; 2024 [cited 2024 Jul 13]. Available from: https://zenodo.org/records/12736756

域代码已更改

域代码已更改
